# Histoid leprosy - A rare clinical presentation^☆^^☆☆^

**DOI:** 10.1016/j.abd.2021.02.003

**Published:** 2021-07-27

**Authors:** Angélica Bauer, Letícia Maria Eidt, Renan Rangel Bonamigo, Renata Heck

**Affiliations:** a Sanitary Dermatology Outpatient Clinic, Health Department of Rio Grande do Sul State, Porto Alegre, RS, Brazil.; b Faculty of Medicine, Universidade Federal do Rio Grande do Sul, Porto Alegre, RS, Brazil.

**Keywords:** Communicable diseases, Leprosy, Leprosy, multibacillary, *Mycobacterium leprae*

## Abstract

Histoid leprosy is considered a rare form of lepromatous leprosy. Its peculiar clinical picture makes its diagnosis a challenging one, which can delay starting treatment and perpetuate the disease as endemic. In addition to representing a reservoir of bacilli, and being an important means of contamination, these patients have greater resistance to standard treatment. This is a report of a typical case of this rare presentation, aiming to share the knowledge and favor earlier diagnosis of the disease.

Histoid leprosy is a rare and highly transmissible variant of lepromatous leprosy.^1^, ^2^, ^3^, ^4^, ^5^, ^6^, ^7^ It presents with erythematous-brownish papules and nodules, with smooth or rarely umbilicated surface.^1^, ^2^, ^3^, ^4^, ^5^, ^6^, ^7^ Due to its unusual clinical features, it is a challenging diagnosis.^1^, ^2^, ^3^, ^4^

This is the report of a 60-year-old male patient, with skin lesions for one year, presenting to a reference service for leprosy management. He denied contact with leprosy. He had keloidiform erythematous-brownish lesions, with a smooth surface, some with umbilication, distributed mainly on the trunk and face (Figure 1, Figure 2). He had a preserved corneal reflex, absence of lagophthalmos, trichiasis, and ectropion. There were no thickened nerves and no muscle strength or sensory alterations in the upper limbs. At the examination of the lower limbs, decreased protective sensitivity in both feet was detected. He had a Grade 1 physical disability. Bacilloscopy showed a mean bacillary index (MBI) of 4.75; with 2% of whole bacilli and clusters. Histopathological analysis showed macrophages with clear, vacuolated cytoplasm, some with phagocytized bacilli and an evident Grenz zone. In the periphery of the lesion, histiocytes in a storiform pattern enclosed collagen fibers and extended into the deep dermis. The Ziehl Neelsen stain showed the presence of multiple bacilli (Figure 3, Figure 4). With the diagnosis of histoid leprosy, multibacillary (MB) multidrug therapy (MDT) was started.

**Figure 1:**
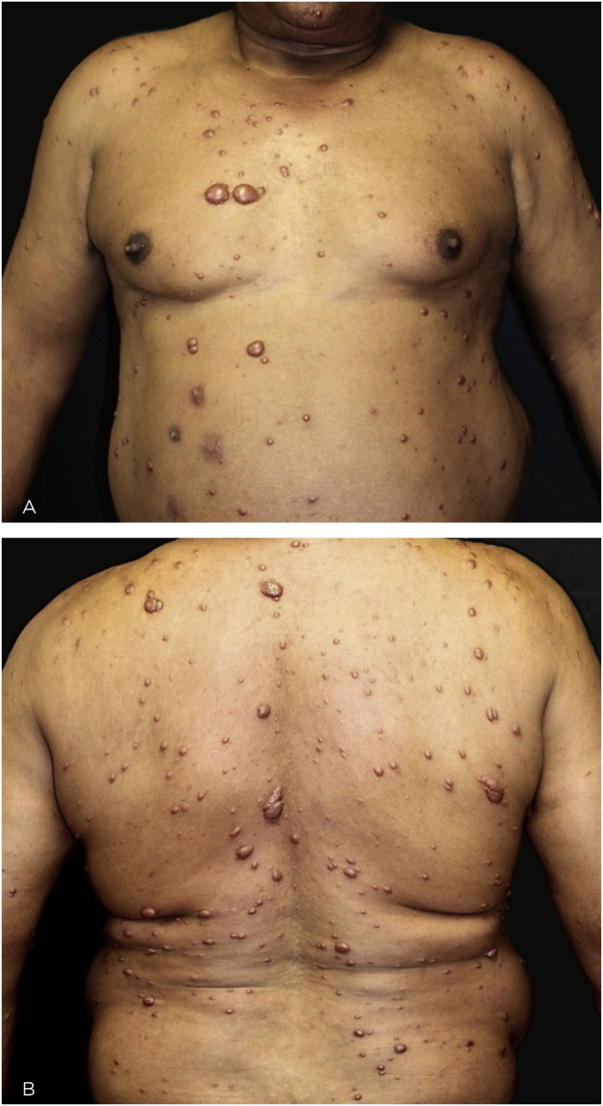
Multiple papules and nodular lesions diffusely distributed on the trunk.

**Figure 2:**
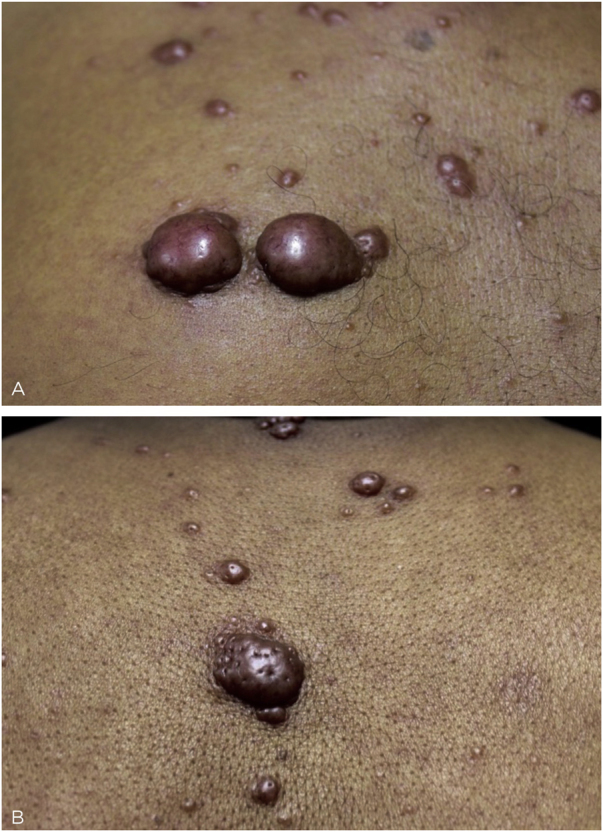
(A), Keloidiform nodular lesions, with smooth surface and an erythematous-brownish color, with evident vessels. (B), Papulonodular lesion with umbilications and satellite umbilicated papules.

**Figure 3:**
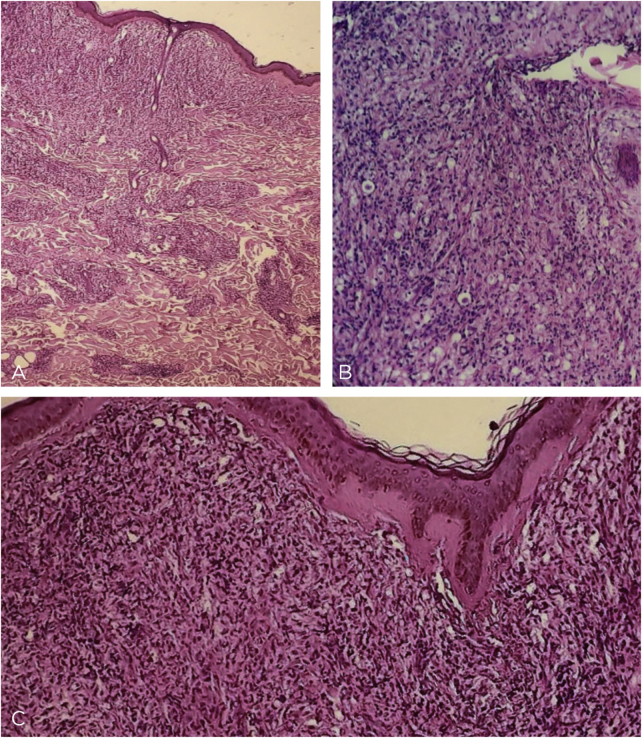
Light microscopy - (A), at low power one sees a Grenz zone; and a lymphohistiocytic infiltrate throughout the dermis, interspersed with collagen fibers (Hematoxylin & eosin, ×40). (B), Vacuolized clear-cytoplasm histiocytes, along with lymphocytic infiltrate (Hematoxylin & eosin, ×200). (C), Detail of the Grenz zone; multiple histiocytes, some of them spindle-shaped in a storiform pattern (multidirectional) (Hematoxylin & eosin, ×100).

**Figure 4:**
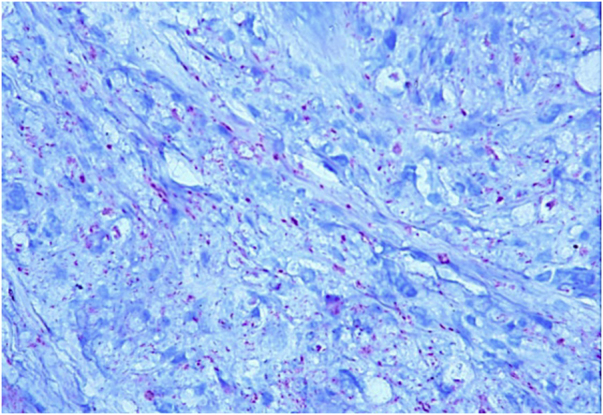
Several bacilli stained with fuchsin, acquiring a pinkish-red color. They are isolated and grouped. (Ziehl-Neelsen, ×40).

At the end of the supervised MB MDT, the patient was reevaluated at the referral center and a new intradermal smear bacilloscopy was performed to control and identify possible recurrence or leprosy reactions.^5^, ^8^, ^9^ Bacilloscopy showed an MBI = 4.25; intact and grouped bacilli.^4^, ^5^, ^8^ When the MDT was stopped, the patient presented a type II leprosy reaction (erythema nodosum leprosum). It was decided, by the reference medical team, to prescribe an additional 12 months of MB MDT.^5^, ^9^

The histoid leprosy subtype has been associated with dapsone resistance and mutations of *Mycobacterium leprae* strains due to inadequate treatment.^1^, ^2^, ^4^, ^7^ Currently, there is an increase in cases of the histoid form again.^1^, ^2^, ^3^, ^4^, ^5^ There is an association with increased cellular and also humoral immunity, absent in lepromatous patients, increasing the number of T lymphocytes locally. However, bacillary hyperactivity and the attempt to contain the infection, end up weakening the local immune system.^1^, ^3^, ^4^, ^6^^,^^7^ The lesions represent a reservoir of *Mycobacterium leprae* and are extremely infectious.^1^, ^2^, ^3^, ^4^, ^5^, ^6^, ^7^

According to previous studies, type II leprosy reactions were not prevalent in histoid patients; however, Brazilian reports show a high incidence of erythema nodosum leprosum.^2^, ^3^ It occurred in the present case, which developed into a type II reaction.

Differential diagnoses include keloids, dermatofibromas, disseminated reticulohistiocytosis, xanthomas, lobomycosis, skin metastases, neurofibromas, lymphomas, angiosarcoma.^2^, ^4^, ^10^

The histopathology of histoid leprosy encompasses three patterns: pure fusocellular, fusocellular with an epithelioid component and fusocellular with vacuolated cells.^2^, ^3^ Bacilli phagocytized by macrophages, isolated bacilli and grouped bacilli were observed.^1^, ^2^, ^6^ The histoid variant may presente an inflammatory cell infiltrate, containing mainly lymphocytes. The spindle-shaped histiocytes are organized in a storiform pattern and enclose collagen fibers and fibroblasts in the periphery of the lesion.^1^, ^2^, ^3^, ^6^ Bacilli stained with Ziehl-Neelsen or Fite-Faraco staining methods can appear in parallel arrangements close to the histiocytes.^2^, ^6^

Despite being a rare form of leprosy, these patients are considered major disease transmitters, thus being of special importance regarding early diagnosis and treatment.^1^, ^2^, ^3^, ^4^

## Financial support

None declared.

## Authors’ contributions

Angélica Bauer: Contributed intellectually to the design and creation of the article; participated in the writing of the manuscript draft; critical and scientific review of the content; approval of the final version of the manuscript.

Letícia Maria Eidt: Contributed intellectually to the design and creation of the article; participated in the writing of the manuscript draft; critical and scientific review of the content; approval of the final version of the manuscript.

Renan Rangel Bonamigo: Contributed intellectually to the design and creation of the article; participated in the writing of the manuscript draft; critical and scientific review of the content; approval of the final version of the manuscript.

Renata Heck: Contributed intellectually to the design and creation of the article; participated in the writing of the manuscript draft; critical and scientific review of the content; approval of the final version of the manuscript.

## Conflicts of interest

None declared.
